# Supplementary figures and images for: Intranasal Delivery of Oncolytic Adenovirus XVir-N-31 via Optimized Shuttle Cells Significantly Extends Survival of Glioblastoma-Bearing Mice
Source: Cancers (Basel). 2023 Oct 10;15(20):4912. doi: 10.3390/cancers15204912 (PMC10605419; doi:10.3390/cancers15204912)

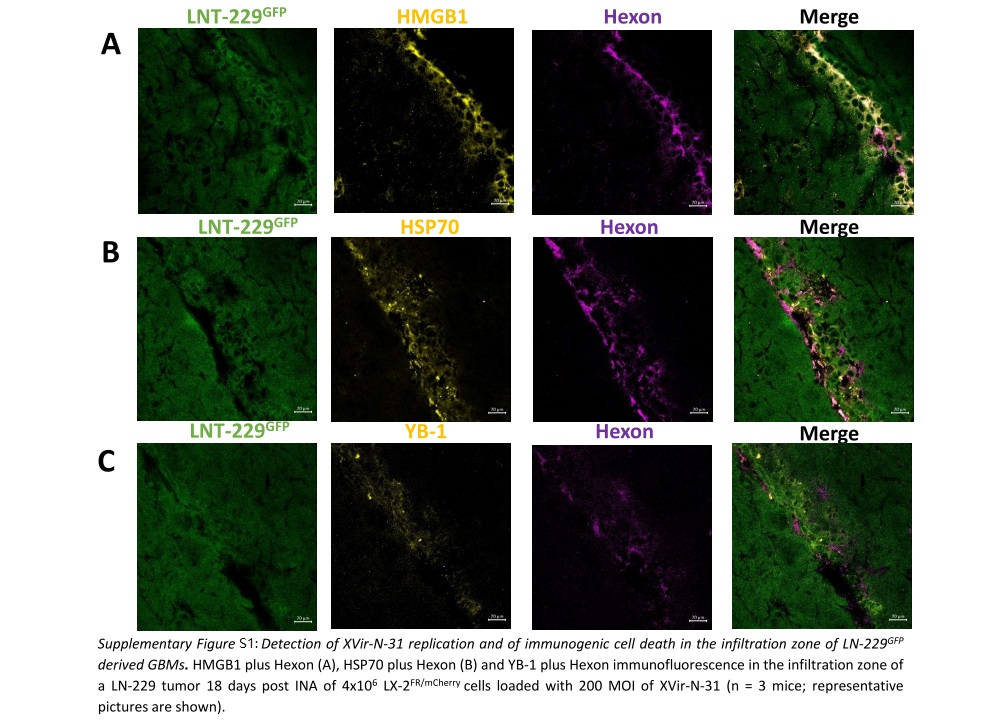

Supplement: Supplementary file 1 [file cancers-15-04912-s001.zip › Figure S1.jpg]

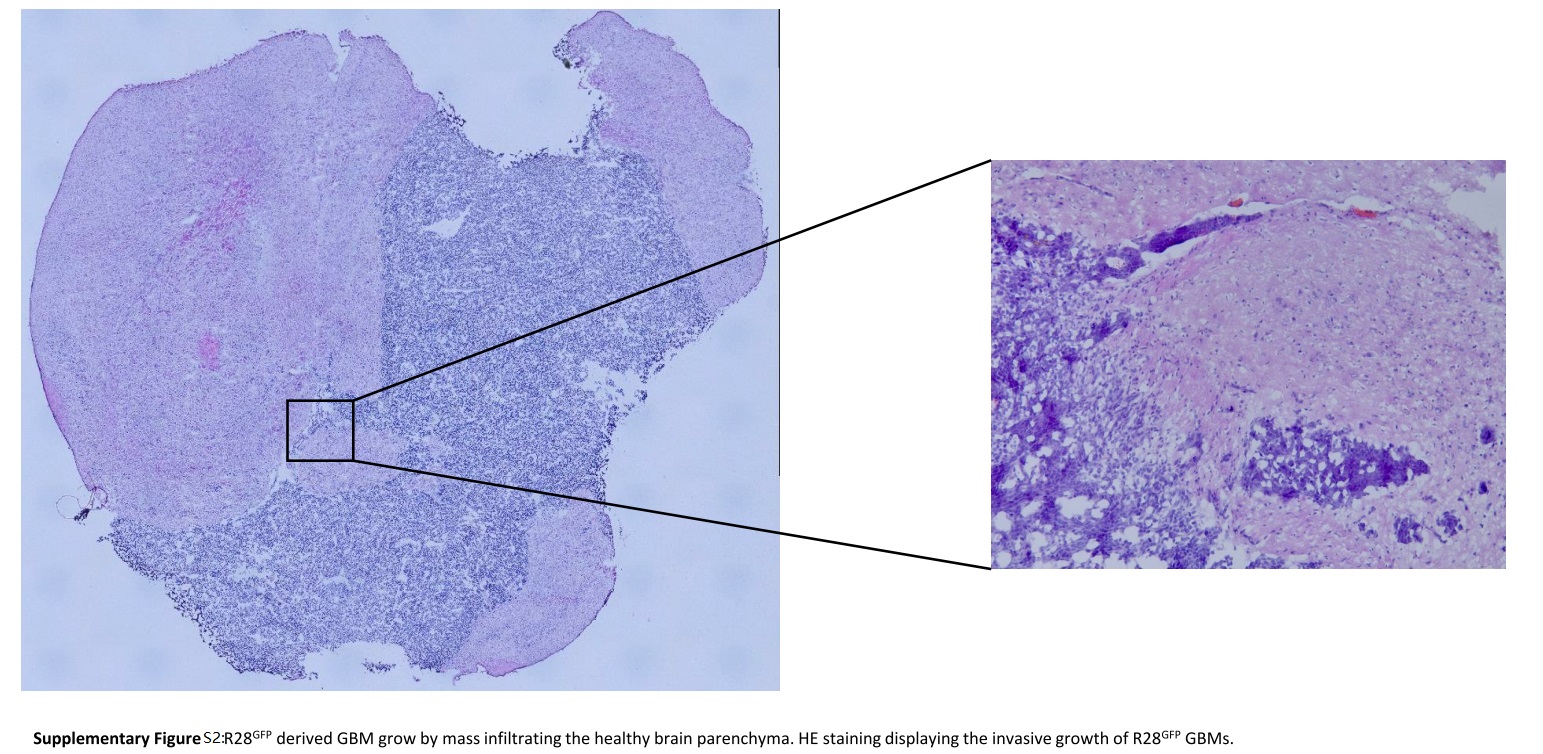

Supplement: Supplementary file 1 [file cancers-15-04912-s001.zip › Figure S2.jpg]

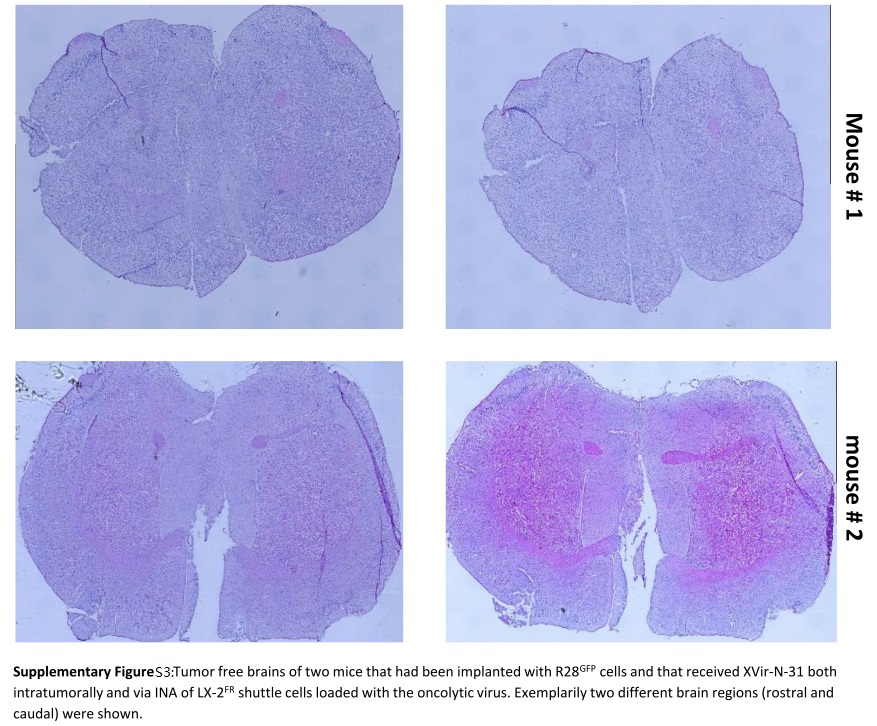

Supplement: Supplementary file 1 [file cancers-15-04912-s001.zip › Figure S3.jpg]

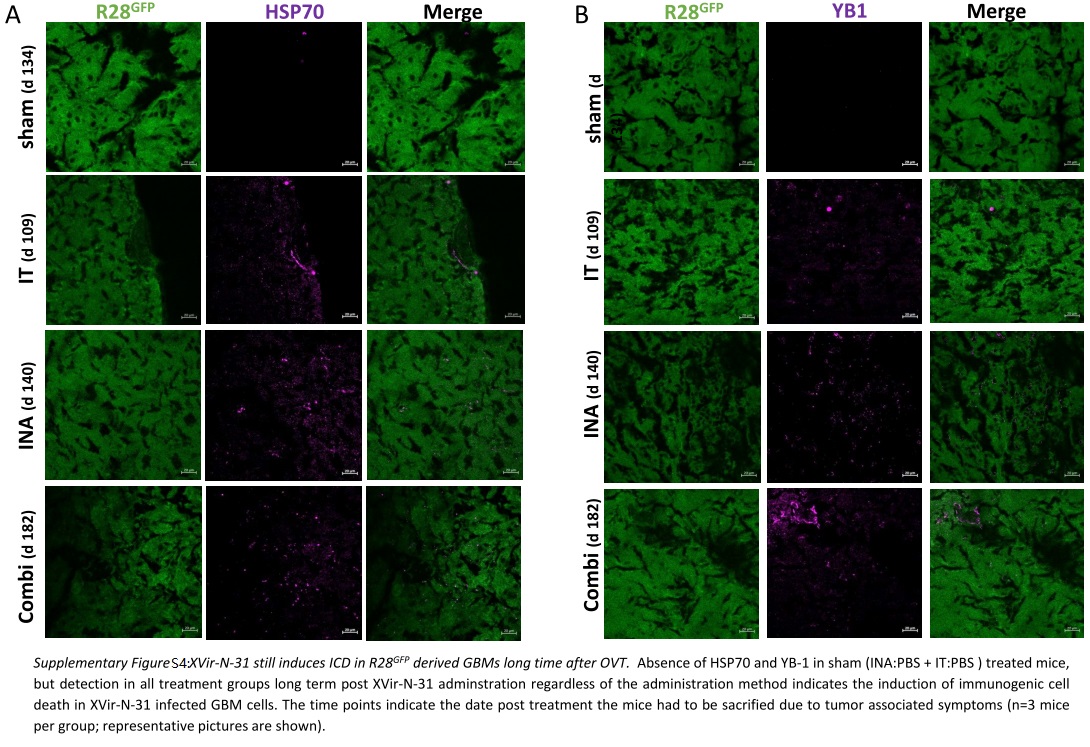

Supplement: Supplementary file 1 [file cancers-15-04912-s001.zip › Figure S4.jpg]

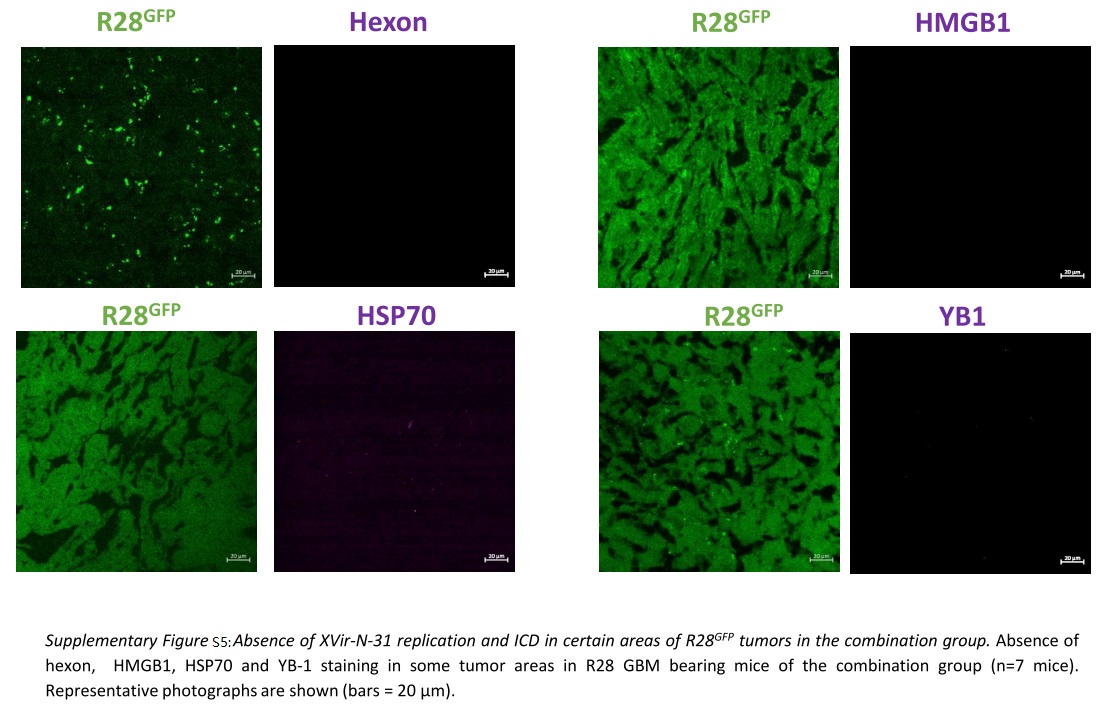

Supplement: Supplementary file 1 [file cancers-15-04912-s001.zip › Figure S5.jpg]

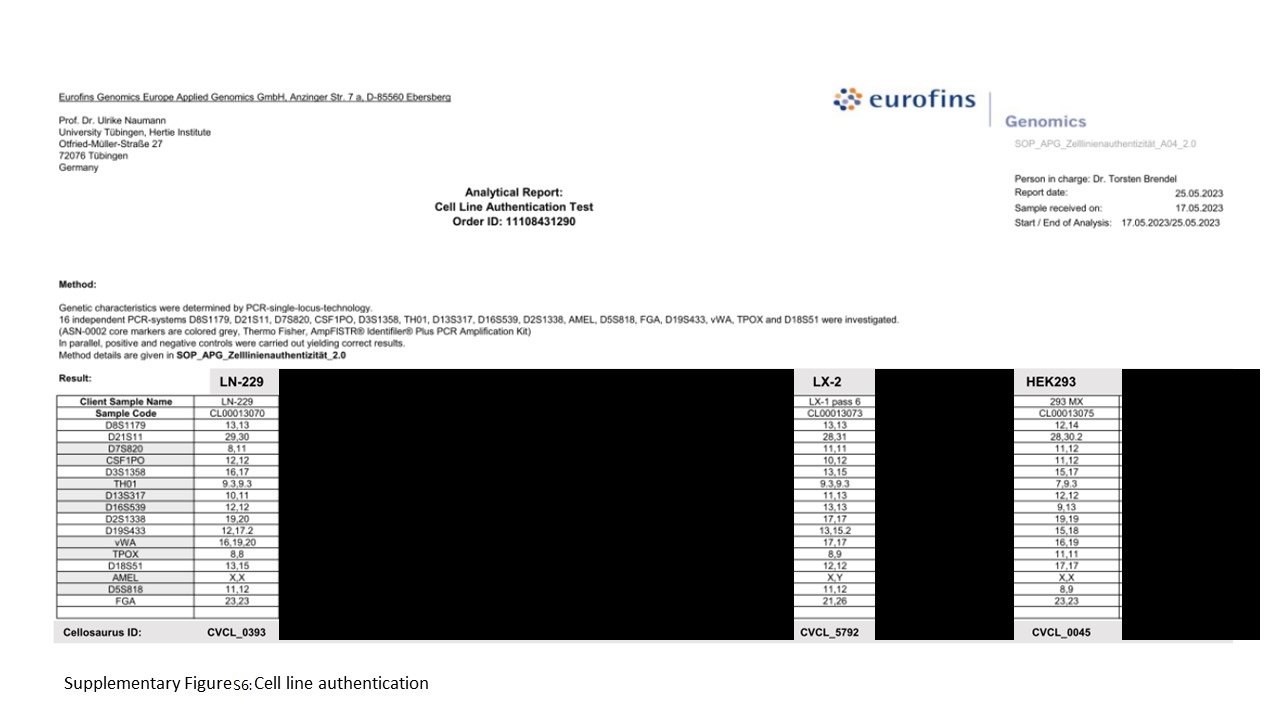

Supplement: Supplementary file 1 [file cancers-15-04912-s001.zip › Figure S6.jpg]
